# The Use of a Commercially Available Endovascular Filter Catheter (Capturex^®^) for Thoracic Endovascular Aortic Repair (TEVAR) in a Patient with a Coral Reef Aorta

**DOI:** 10.1007/s00270-022-03147-6

**Published:** 2022-04-26

**Authors:** Rupert Horst Portugaller, Jakob Steiner, Florian Schmid, Igor Knez, Martin Asslaber, Hannes A. Deutschmann

**Affiliations:** 1 Division of Neuroradiology, Vascular and Interventional Radiology, Department of Radiology, Medical University of Graz (MUG), Auenbruggerplatz 9A, A-8036 Graz, Austria; 2 Division of General Radiological Diagnostics, Department of Radiology, Medical University of Graz (MUG), Auenbruggerplatz 9A, A-8036 Graz, Austria; 3 Division of Cardiac Surgery, Department of Surgery, Medical University of Graz (MUG), Auenbruggerplatz 29/3, A-8036 Graz, Austria; 4 Department of Diagnostic and Scientific Pathology, Medical University of Graz (MUG), Neue Stiftingtalstraße 6, A-8036 Graz, Austria

**Keywords:** Coral reef aorta, TEVAR, Filter catheter, Thoracic aortic aneurysm

## Abstract

Due to the risk of mobilizing plaque fragments, transfemoral TEVAR is a potentially dangerous procedure in patients with a coral reef aorta. We describe a practical method for transfemoral TEVAR in a patient with a degenerative thoracic aneurysm and a coral reef aorta. After placing a filter catheter in the abdominal aorta via a contralateral percutaneous femoral access, a working channel through the distal thoracic aorta was created with a balloon-expandable stent graft in the coral reef segment. Thereafter, transfemoral TEVAR could be performed successfully, without any complications. The additional use of a percutaneously placed filter catheter potentially allows reduction of peripheral embolism and hence may prevent patients from more invasive treatment.

## Introduction

A so-called coral reef aorta typically contains extensive calcified plaques that protrude far into the vessel lumen [1–3]. Hence, endovascular manipulations in such a vessel bear a certain risk of releasing emboli. We describe the use of an intravascular filter catheter for transfemoral thoracic endovascular aortic repair (TEVAR) of a degenerative aneurysm in a patient with a coral reef aorta.

## Case Report

A 60-year-old woman presented to a tertiary medical center with dry cough and upper back pain for one week. Long-term arterial hypertension was known, but recently, difficult to control with her actual twofold antihypertensive medication. At admission, arterial blood pressure was 185/95 mmHg. She had no past medical history of nicotine consumption, diabetes or other additional vascular risk factors.

**Fig. 1:**
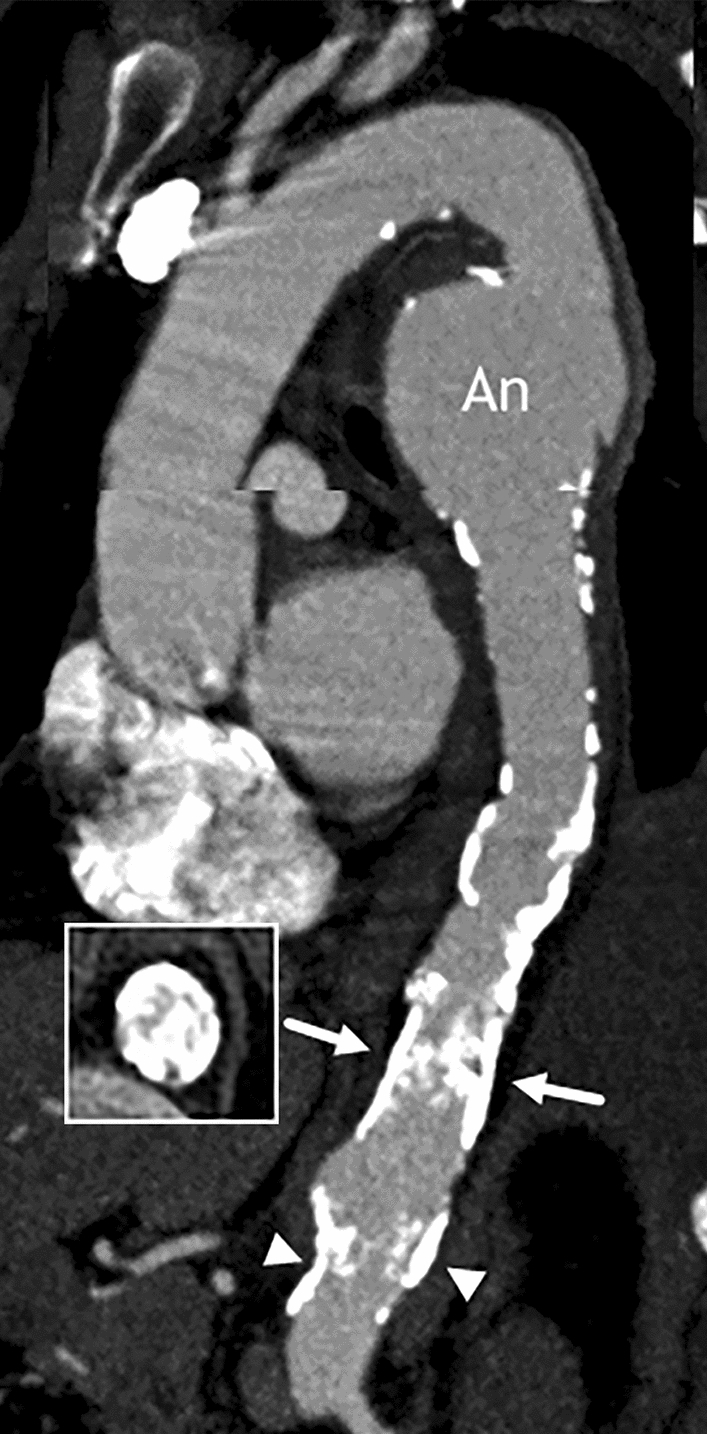
Pre-interventional CTA (composed reformatted images): Five-cm-wide false aneurysm of the proximal descending thoracic aorta (An). Obstructive wall calcifications at the aortic hiatus (arrows). Nonobstructive calcifications in the suprarenal abdominal aorta (arrowheads)

CT angiography (Fig. 1) revealed a 5-cm-wide, saccular aneurysm of the proximal descending thoracic aorta. The distal thoracic and the suprarenal abdominal aorta were affected by extensive wall calcifications that extended far into the lumen, typical for a coral reef aorta. At the distal thoracic segment, the protruding plaques formed a near-occlusive plug. The celiac trunk and superior mesenteric artery revealed no significant luminal stenoses. The renal ostia were not affected by the calcifications and, also free of lumen alterations.

Due to the possible causal connection with the patient´s upper back pain, early treatment of the aneurysm was scheduled. To act as minimally invasive as possible, transfemoral TEVAR was attempted. For the procedure, a filter catheter should be placed in the abdominal aorta to protect from eventual embolism. Then, a balloon-expandable stent graft should be deployed at the near-occlusive coral reef aortic segment to create a working channel for the introduction of an aortic endoprosthesis.

**Fig. 2:**
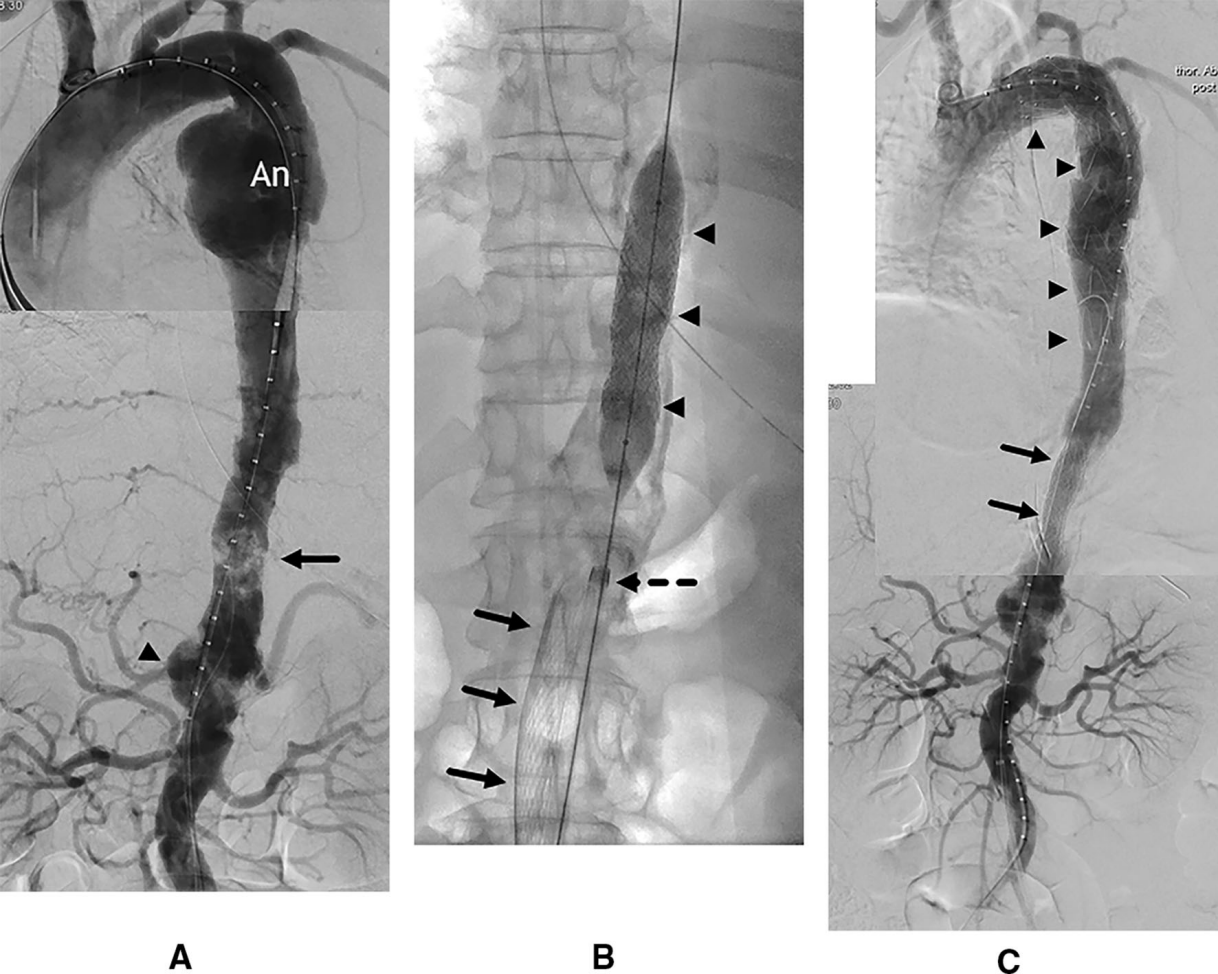
Peri-interventional digital subtraction angiographies (composed series): Obstructive plug-forming calcifications (A, arrow). Atherosclerotic ulceration at the level of the celiac trunk (A, arrowhead). Via the ipsilateral 11 F sheath (B, dotted arrow), deployment of the BeGraft^®^ at the obstructive segment (B, arrowheads). Open Capturex^®^ filter (B, arrows). Complete seal of the false aneurysm as well as free flow through the thoracic endoprosthesis (C, arrowheads) and the BeGraft^®^ (C, arrows)

**Fig. 3:**
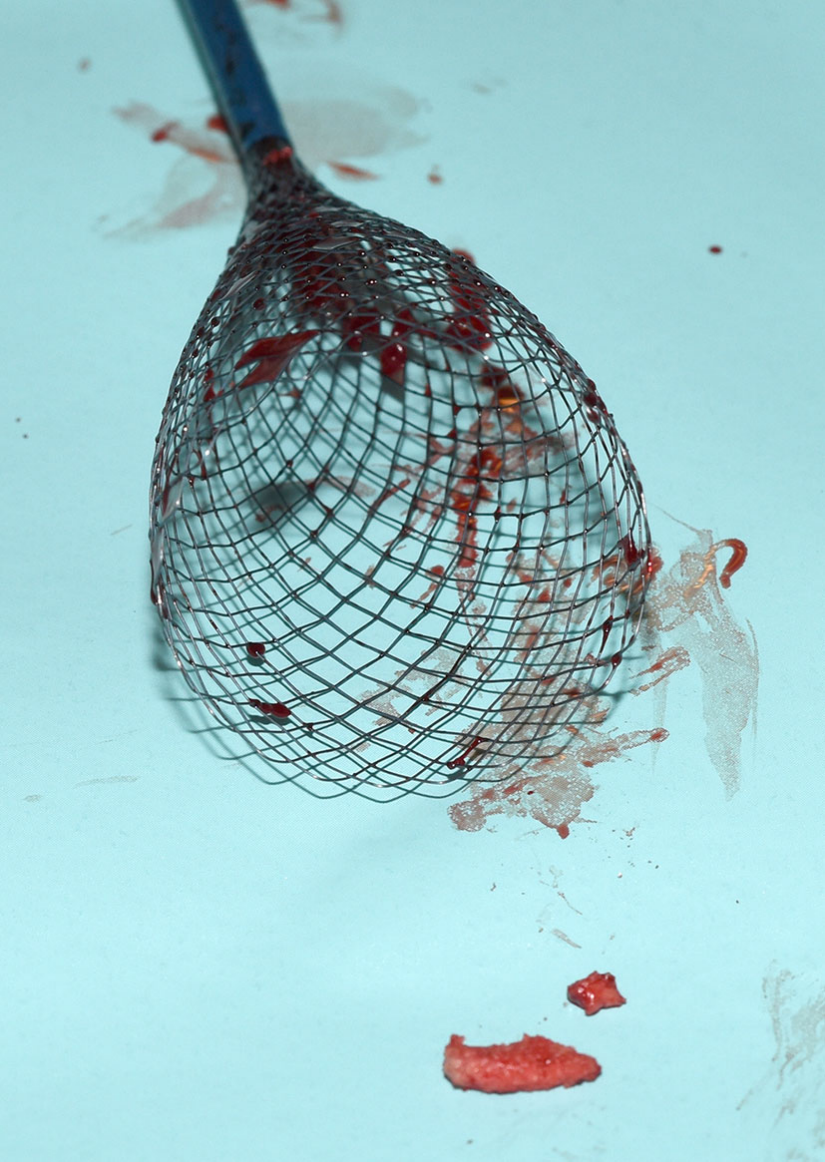
Capturex^®^ filter catheter and the caught calcified plaque particles

**Fig. 4:**
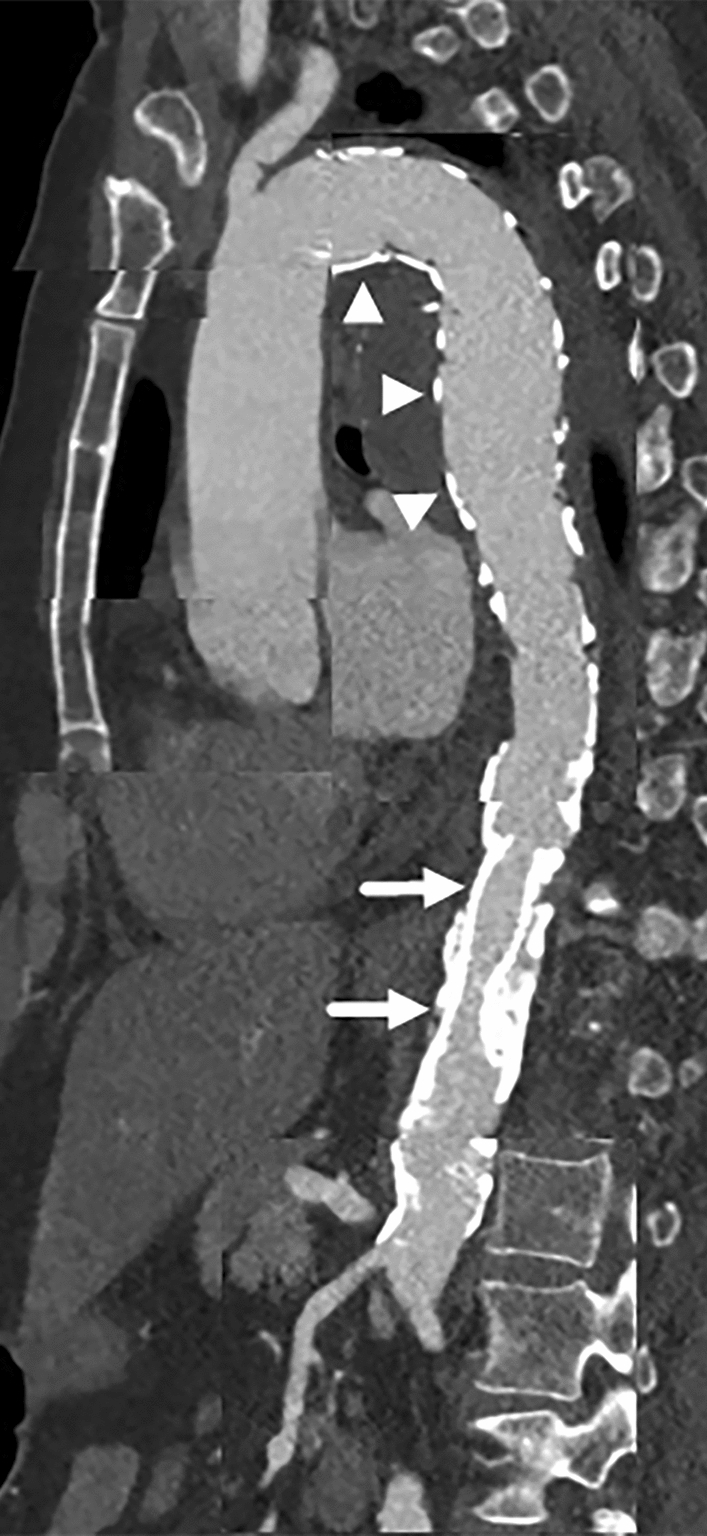
CT-angiography 6 days after intervention (composed reformatted images): Complete occlusion of the false aneurysm by the aortic endoprosthesis (arrowheads). Patent BeGraft® (arrows)

Perioperatively, the patient received intravenous antibiotic therapy with 3 × 1 g meropenem. Intravenous heparin was also given by the anesthesiologist. In general anesthesia, via an open surgical right femoral access a hydrophilic guidewire and a pigtail angiography catheter were cautiously advanced into the ascending aorta. After digital subtraction angiography (DSA, Fig. 2A), an 11-French sheath was placed just below the coral reef segment. Then a 10-French sheath of the filter catheter system was introduced via a percutaneous left groin access. The sheath was carefully advanced over a stiff guide wire until its tip was just below the obstructive calcifications. Then, the filter catheter (Capturex^®^, Straub Medical AG, Wangs, Switzerland) was introduced and deployed immediately distal to the obstructive coral reef section. In this way, the filter covered all visceral branches of the abdominal aorta. Further, a balloon-expandable 16/59 mm BeGraft^®^ aortic stent graft (Bentley InnoMed GmbH, Hechingen, Germany) was introduced via the ipsilateral 11-French sheath and deployed at the obstructive coral reef aortic segment (Fig. 2B) [4]. Thus, having created a safe working channel, a 32/164 mm Relay^®^ PRO thoracic stent graft (Bolton Medical, Inc., Sunrise, Fl, U.S.A.) was introduced via the right common femoral artery and deployed over the aneurysm. After a control angiogram (Fig. 2C), the filter catheter could be collapsed and removed percutaneously without significant resistance. Upon inspection, some solid fragments were found inside the filter that were histologically classified as up to 12 × 5 mm calcified plaques (Fig. 3). The right femoral arterial access was closed surgically. At the left puncture site, hemostasis was achieved by a percutaneous suture device (Prostar^TM^XL Percutaneous Surgical Vascular System, Abbott Vascular Inc. Santa Clara, CA, USA). After completion of the procedure, the patient was transferred to the intensive care unit for further surveillance. She did not develop any symptoms of central, visceral or peripheral embolism. Heparin was continued for 48 h; daily oral clopidogrel, 75 mg, for 3 months and aspirin 100 mg, indefinitely, were also prescribed. Due to ongoing hypertensive episodes, the patient’s medication had to be adjusted. A control CT angiography six days after intervention showed complete thrombosis of the aneurysm sac, an open stent graft in the coral reef segment as well as free perfusion of the visceral arteries (Fig. 4). Finally, she was discharged without complaints.

## Discussion

Transfemoral TEVAR is usually the treatment of choice for patients with aneurysms in the descending thoracic aorta. However, this current case shows that the coral reef calcification posed a certain obstacle for an aortic endoprosthesis. Since any unprotected manipulation in a coral reef aorta may be vicious by mobilizing plaque fragments, an endovascular safety filter was placed first. This Capturex^®^ filter catheter consists of a self-expanding nitinol filter basket on a wire-reinforced catheter shaft. It is inserted via a 10-French sheath and can be deployed or re-captured by moving the sheath. As a temporary filter, it was primarily designated to catch thrombi in the vena cava, but it can also be used in the aorta [5]. Secondly, a safe access route was generated for the aortic endoprosthesis, by placing of a balloon-expandable stent graft over the protruding calcified plaques. When we passed the introducer system of the aortic endoprosthesis along the deployed filter, the filter basket was likely to be pushed aside from the aortic wall. However, the patient’s lack of embolic symptoms indicates that this loss of filter alignment was not of relevance.

Two similar cases with filter protection during TEVAR have been published. In the first study, a large Wallstent was introduced via a surgical femoral access, kept partially opened during the procedure and re-captured and withdrawn thereafter [6]. In the other case, a homemade intra-aortic filter device was created with a braided nitinol basket where a polyester fabric was embedded. Through a 12-F sheath, it was also inserted and removed via an open surgical femoral access [7]. Both procedures were performed successfully. The technique with the partially opened Wallstent seems to be quite practical. However, when deployed too extensively, a minimum risk of losing the Wallstent may exist. The second technique requires some more handwork and thus appears more inconvenient. In contrast to these techniques, the filter catheter that we used is commercially available and can be applied percutaneously.

## Conclusion

In the presented case, the use of a filter catheter allowed endovascular manipulations for TEVAR in a thoracoabdominal coral reef aorta without complications. Hence, it features a valuable tool to prevent patients from more invasive alternative access methods.
